# The Reciprocal Space Station Forum for Structural Scientists

**DOI:** 10.1063/4.0001155

**Published:** 2025-10-27

**Authors:** Doris Mai, Kara A Zielinski, Kevin M Dalton

**Affiliations:** 1 Machine Learning and Computer Vision Group, LCLS Data Systems, SLAC National Accelerator Laboratory, Menlo Park, CA, USA; 2 School of Applied and Engineering Physics, Cornell University, Ithaca, NY, USA

## Abstract

Announcing a new web forum for structural biologists! The RS-Station Forum (https://discourse.rs-station.org) is a modern online community focused on data analysis and education. On the RS-Station, you can ask questions to developers and experts while enjoying features such as markdown formatting, code syntax highlighting, and mathematical typesetting. Based on the popular, open-source Discourse webapp, the forum benefits from a vibrant ecosystem of plugins. Stop by the poster to learn more about ways to participate!

